# Quantitative collateral score for the prediction of clinical outcomes in stroke patients: Better than visual grading

**DOI:** 10.3389/fnins.2022.980135

**Published:** 2022-10-25

**Authors:** Qingqing Lu, Haiyan Zhang, Xin Cao, Junyan Fu, Yuning Pan, Xiaodong Zheng, Jianhong Wang, Daoying Geng, Jun Zhang

**Affiliations:** ^1^State Key Laboratory of Medical Neurobiology, Department of Radiology, Huashan Hospital, Fudan University, Shanghai, China; ^2^Department of Radiology, Ningbo First Hospital, Ningbo, China; ^3^Department of Neurology, Huashan Hospital, Fudan University, Shanghai, China; ^4^National Center for Neurological Disorders, Shanghai, China; ^5^Center for Shanghai Intelligent Imaging for Critical Brain Diseases Engineering and Technology Research, Huashan Hospital, Fudan University, Shanghai, China

**Keywords:** quantitative collateral score, visual collateral score, acute ischemic stroke, mechanical thrombectomy, low-density lipoprotein cholesterol, independent prognostic predictor

## Abstract

**Objectives:**

To identify preoperative prognostic factors for acute ischemic stroke (AIS) patients receiving mechanical thrombectomy (MT) and compare the performance of quantitative collateral score (qCS) and visual collateral score (vCS) in outcome prediction.

**Methods:**

Fifty-five patients with AIS receiving MT were retrospectively enrolled. qCS was defined as the percentage of the volume of collaterals of both hemispheres. Based on the dichotomous outcome assessed using a 90-day modified Rankin Scale (mRS), we compared qCS, vCS, age, sex, National Institute of Health stroke scale score, etiological subtype, platelet count, international normalized ratio, glucose levels, and low-density lipoprotein cholesterol (LDL-C) levels between favorable and unfavorable outcome groups. Logistic regression analysis was performed to determine the effect on the clinical outcome. The discriminatory power of qCS, vCS, and their combination with cofounders for determining favorable outcomes was tested with the area under the receiver-operating characteristic curve (AUC).

**Results:**

vCS, qCS, LDL-C, and age could all predict clinical outcomes. qCS is superior over vCS in predicting favorable outcomes with a relatively higher AUC value (qCS vs. vCS: 0.81 vs. 0.74) and a higher sensitivity rate (qCS vs. vCS: 72.7% vs. 40.9%). The prediction power of qCS + LDL-C + age was best with an AUC value of 0.91, but the accuracy was just increased slightly compared to that of qCS alone.

**Conclusion:**

Collateral scores, LDL-C and age were independent prognostic predictors for patients with AIS receiving MT; qCS was a better predictor than vCS. Furthermore, qCS + LDL-C + age offers a strong prognostic prediction power and qCS alone was another good choice for predicting clinical outcome.

## Introduction

The use of mechanical thrombectomy (MT) has increased dramatically since several large randomized clinical trials have confirmed that neurological outcomes of patients with acute ischemic stroke (AIS) were better when treated with MT than when treated with other methods (Albers et al., 2018; Nogueira et al., 2018; Powers et al., 2019). However, in clinical practice, the clinical outcome remains unsatisfying with higher rate of moderate to severe disability at discharge (79%) and higher 3-month mortality (29%) (Wollenweber et al., 2019; Qureshi et al., 2020). In addition, the cost of MT can be prohibitively expensive; therefore, it is particularly important to identify preoperative factors for the prediction of postoperative outcome in patients eligible for MT treatment; this shall help to avoid over-treatment as well.

Previous studies have shown that collateral circulation plays an important role in the prognosis of patients with AIS, and patients with good collaterals have a high rate of functional independence (Berkhemer et al., 2016; Lu et al., 2019). However, de Havenon et al. (2019) reported that collateral circulation only affected the infarct core volume and infarct rate and was not a predictor of neurological prognosis or mortality after MT. Collateral circulation was most commonly evaluated by manually performed visual grading of computed tomography angiography (CTA) images; these findings are reflected as the visual collateral score (vCS). This method requires experienced neuroradiologists and has frequent intra-observer disagreement (McVerry et al., 2012; Cui et al., 2021). Such inaccurate methods may limit the prognostic efficacy of collateral circulation, and a reliable quantitative collateral score (qCS) method is needed (Shi et al., 2019). Furthermore, the robustness of qCS in indicating the prognosis of patients with AIS receiving MT remains unclear. Low-density lipoprotein cholesterol (LDL-C) is known as bad cholesterol and is associated with increased inflammatory response, which reflects as increased incidences of vascular diseases and worsened stroke outcomes (Kim and Cho, 2021). Therefore, whether LDL-C can predict the stroke outcome needs to be investigated.

In this study, we quantified the collateral volume and acquired qCS using the ITK-SNAP software. The study was aimed at identifying preoperative prognostic factors for patients with AIS receiving MT, and demonstrating whether collaterals assessed by qCS could predict clinical outcomes better than vCS.

## Materials and methods

### Study population

From January 2018 to May 2021, we retrospectively selected consecutive patients who presented with symptoms of AIS within 24 h of onset and those who underwent MT from our single-institution stroke center. This study was reviewed and approved by the local institutional review board, and the need for written informed consent was waived owing to the retrospective design of the study and anonymization of the data. The inclusion criteria were as follows: (1) patient age > 18 years; (2) premorbid modified Rankin Scale (mRS) score ≤ 2; (3) occlusions in the M1 segment of the middle cerebral artery (MCA) and/or intracranial internal carotid artery (ICA); and (4) preinterventional computed tomography perfusion (CTP) performed for patients within 6–24 h from the time last known well with an MIStar-determined ischemic core volume < 70 mL, mismatch ratio ≥ 1.8, and mismatch volume ≥ 15 mL. The exclusion criteria were as follows: (1) intracranial hemorrhage identified by non-contrast computed tomography (NCCT); (2) a low-density area greater than 1/3rd of the MCA territory on NCCT; (3) occlusion of other intracranial arteries or stenosis of MCA; (4) a history of a moderate to large stroke in the contralateral hemisphere resulting in a measurable decrease in vasculature; and (5) CTA or CTP images with obvious motion artifacts or improper phase; (6) Moyamoya disease; (7) incomplete clinical data.

### Image acquisition

Patients with symptoms of AIS underwent the imaging protocol according to the early patient management guidelines (Powers et al., 2019). CTP was not required for selecting suitable patients within 6 h from onset but was required for selecting suitable patients within 6–24 h from onset. Images were obtained using a 64-multislice CT scanner (Discovery CT750 HD, GE Healthcare) in the order of NCCT, CTP, and CTA. Single-phase CTA was performed during the administration of 80 mL of non-ionic iodine contrast agent (ioversol 320 mgI/mL, Jiangsu Hengrui Medicine Co., Ltd., China) at the rate of 4 mL/s, followed by the administration of 40 mL of saline at the same rate. The parameters were as follows: tube voltage 140 kVp, tube current 630 mA, rotation time 0.5 s, and slice thickness 0.625 mm; the area covered was from the aortic arch to the top of the cranium. CTP was obtained using volume shuttle scanning started with a 5–10-s delay after contrast adjustment. Acquisition parameters for CTP were tube voltage 100 kVp, tube current 400 mA, rotation time 0.4 s, and slice thickness 5 mm; the area covered was from the base to the top of the skull.

### Image analysis

**FIGURE 1:**
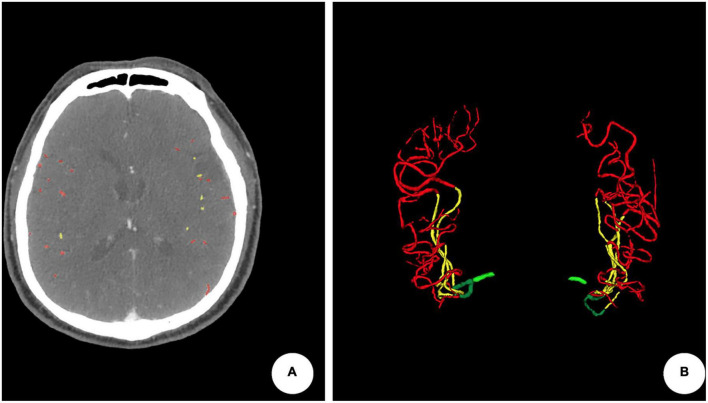
An example of quantitative collateral scoring using the ITK-SNAP software. **(A)** Segmentation of arteries in the middle cerebral artery territories using different labels. The quantitative collateral score was 98.74%. **(B)** 3D representation of the segmented vasculature with occlusion of the left M1 segment.

Axial, coronal, and sagittal 20-mm-thick maximum intensity projection images were reformatted from CTA images. Two experienced neuroradiologists who were blinded to the clinical and CTP data assessed the vCS independently using a previously reported, predefined scoring system (Tan et al., 2007). For disputed cases, an experienced senior neuroradiologist who was also blinded to the data made the final decision. Considering the contralateral side as the reference, the scoring system was divided into four points according to the filling proportion of collaterals in the occluded territory: 0 = absent collaterals (0%), 1 = poor collateral circulation (≤ 50%), 2 = moderate collateral circulation (50--100%, not including 100%), and 3 = good collateral circulation (100%). qCS was defined as the percentage of the volume of collaterals in the occluded area divided by the volume of vessels in the corresponding area on the unaffected side. The vessel volume was obtained by manual segmentation on CTA images using the open-source software ITK-SNAP (version 3.8.0),^1^ and the annotation was performed by the first author of this paper and another experienced neuroradiologist under the supervision of the senior neuroradiologist. Different segmental arteries in the MCA territories were labeled by user-defined colors based on the anatomy (Shapiro et al., 2020). Then, the vessel volume of each segment was automatically calculated (Figure 1). The M3-4 segment and pial collaterals share the same label “M3-distal,” and qCS (M3-distal) represents qCS of the segment of M3 to pial collaterals. The qCS of the whole vessel was presented as qCS (total). The segmental volume and qCS were compared. A commercial software, MIStar (Apollo Medical Imaging Technology, Melbourne, Australia), was used to obtain CTP parameters, including infarct core, ischemic volume, mismatch volume (ischemic volume–infarct core), and mismatch ratio (ischemic volumes/infarct core).

### Clinical evaluation

Baseline clinical data were collected by a neurologist specializing in stroke. Age, sex, admission National Institute of Health stroke scale (NIHSS) score, etiological subtype [trial of ORG 10172 in acute stroke treatment (TOAST) classification], and related laboratory markers (platelet, international normalized ratio, glucose levels, and LDL-C) on admission were recorded. Reperfusion was rated according to the modified Treatment In Cerebral Ischemia (mTICI) score (Goyal et al., 2014) during operation, and successful reperfusion was defined as an mTICI score of ≥ 2b. The patients were divided into two groups according to their 90-day clinical outcome: the favorable outcome group (mRS ≤ 2) and the unfavorable outcome group (mRS > 2).

### Statistical analysis

Statistical analysis was performed using the IBM SPSS Statistics software (Version: 26.0.0.0). The Shapiro–Wilk test was used for normality test. Continuous variables were presented as mean ± standard deviation or median (interquartile range). Categorical variables were expressed as frequency and percentage. Spearman correlation coefficients and intraclass correlation efficient (ICC) were performed to compare qCS and vCS. The Chi-square or Fisher’s exact test and the independent samples *t*-test or Mann–Whitney *U*-test was used to compare the differences between favorable and unfavorable outcome groups. Binary logistic regression analysis was performed to determine the effect on the clinical outcome. The variables with *P* < 0.1 at univariable analysis were included in the multivariable logistic regression analysis. Since both qCS and vCS measured the same factor, they were, respectively, put into the model with other statistically significant parameters. Receiver operating characteristic (ROC) analysis was performed to test the performance of qCS, vCS, and their combination with cofounders in determining favorable outcomes. A *P*-value of < 0.05 was considered statistically significant.

## Results

### Cohort characterization

**FIGURE 2:**
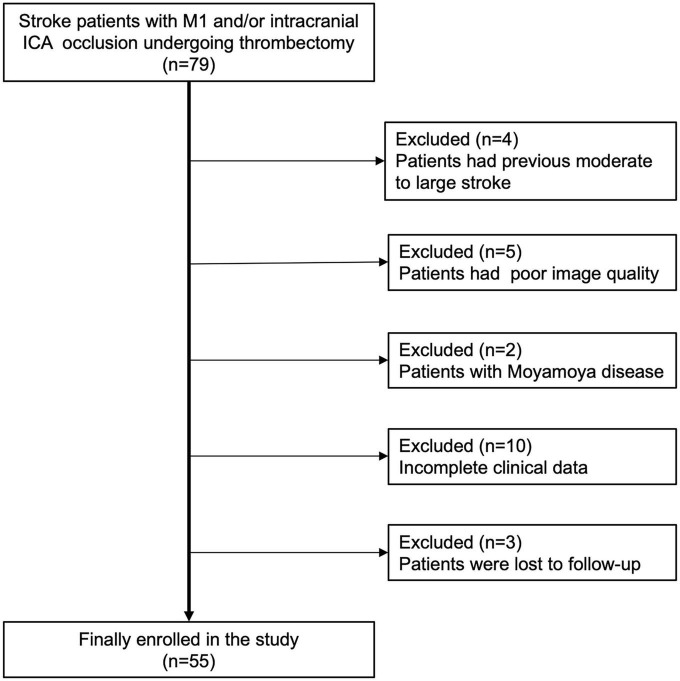
Flow chart of enrolled patients. ICA, internal carotid artery.

A total of 55 patients (median age, 74 years; 38 men) with acute M1 and/or ICA occlusion who underwent MT were enrolled (Figure 2). The median baseline NIHSS score of the patients was 13; 41 patients were evaluated as having moderate-to-good collateral circulation by vCS (Tan score ≥ 2). Herein, 22/55 patients belonged to the favorable outcome group, and 33/55 patients belonged to the unfavorable outcome group; among these 33 patients, 25 had to live dependently and 8 died. The clinical characteristics of all enrolled patients are presented in Table 1. The comparison reveals that patients in the unfavorable outcome group were significantly older and had higher baseline NIHSS score, higher glucose levels, lower LDL-C levels, and worse collateral circulation than patients in the favorable outcome group (Table 1).

**TABLE 1:** Patient characteristics.

Characteristics	All	mRS 0–2 (*n* = 22)	mRS 3–6 (*n* = 33)	*P-*value
Age, years	74 (60–79)	66 ± 12	75 (69–81)	0.013*
Male sex	38 (69.09)	15 (68.18)	23 (69.70)	0.905
TOAST type, LAA/CE	24 (43.64)/22 (40.00)	13 (59.09)/6 (27.27)	12 (36.36)/15 (45.45)	0.245
Baseline NIHSS	13 (10–17)	11 (9–15)	14 (11–20)	0.033*
Platelet, 10^9^/L	193 (172–220)	209 ± 38	183 (161–212)	0.092
INR	1.02 (0.97–1.08)	1.02 (0.98–1.06)	1.03 (0.97–1.14)	0.648
Glucose, mmol/L	7.00 (5.90–8.80)	6.35 (5.68–8.18)	7.40 (6.45–9.40)	0.033*
LDL-C, mmol/L	2.62 ± 0.77	2.92 ± 0.77	2.42 ± 0.71	0.016*
Reperfusion	39 (70.91)	18 (81.82)	21 (63.64)	0.146
mRS score	4 (2–5)	1 (0–2)	5 (4–6)	–
vCS (Tan score)	2 (1–2)	2 (2–3)	2 (1–2)	0.001*
Tan score = 1	14 (25.45)	1 (4.54)	13 (39.39)	–
Tan score = 2	28 (50.91)	12 (54.55)	16 (48.49)	–
Tan score = 3	13 (23.64)	9 (40.91)	4 (12.12)	–
qCS (total), %	71.29 ± 21.20	80.31 ± 18.47	65.28 ± 21.01	0.009*
qCS (M1), %	50.27 (18.64–86.88)	47.67 (16.54–77.70)	50.27 (26.83–92.81)	0.536
qCS (M2), %	60.12 ± 30.98	74.48 (28.13–94.86)	51.16 (36.57–75.51)	0.471
qCS (M3-distal), %	79.10 ± 23.63	93.67 ± 16.73	69.39 ± 22.70	<0.001*
Infarct core, mL	11.00 (4.00–23.00)	8.50 (4.00–18.93)	14.00 (4.50–23.50)	0.144
Ischemic volume, mL	95.34 ± 50.67	83.60 (45.65–114.50)	97.22 ± 44.42	0.434
Mismatch volume, mL	69.00 (46.00–103.00)	65.00 (42.13–108.13)	71.00 (49.50–96.50)	0.810

Continuous variables were presented as mean ± standard deviation or median (interquartile range). Categorical variables were expressed as frequency and percentage. Successful reperfusion was identified as Modified Treatment in Cerebral Ischemia Scale score ≥ 2b.

*Statistically significant.

TOAST, trial of ORG 10,172 in acute stroke treatment; LAA, large-artery atherosclerosis; CE, cardioembolism; NIHSS, National Institutes of Health Stroke Scale; INR, international normalized ratio; LDL-C, low-density lipoprotein cholesterol; mRS, modified Rankin Scale; vCS, visual collateral score; qCS, quantitative collateral score.

### Agreement between visual collateral score and quantitative collateral score

**FIGURE 3:**
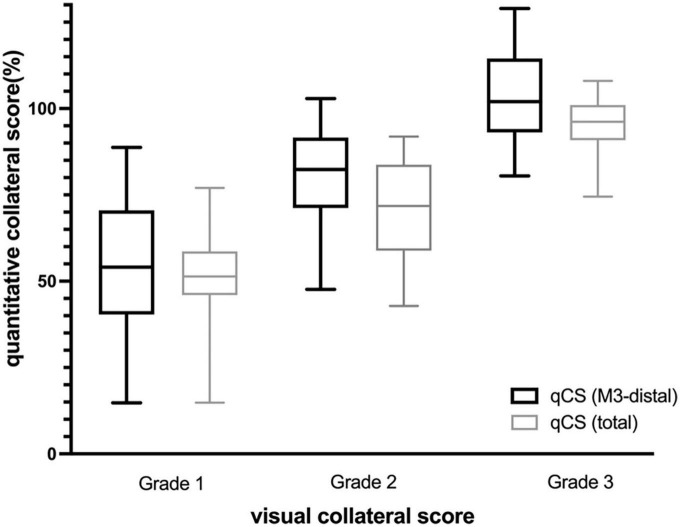
Distribution of qCS per visual collateral score (Box- and-whisker plot). qCS, quantitative collateral score.

**FIGURE 4:**
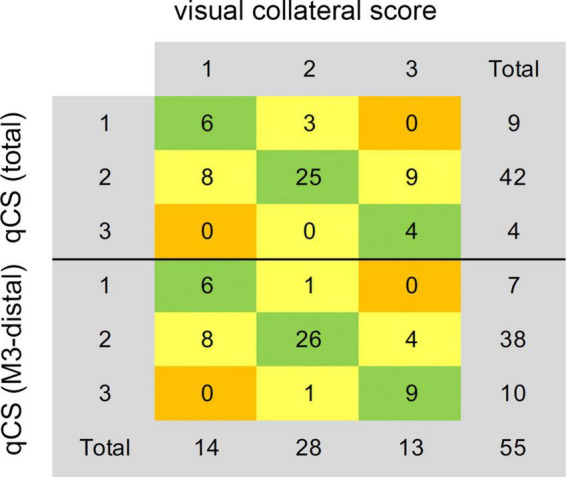
Agreement of the consensus visual collateral score (Tan score) with qCS (total) and qCS (M3-distal). qCS was divided into four points according to the classification criteria of vCS. qCS, quantitative collateral score.

The distribution of qCS (total) and qCS (M3-distal) per vCS is presented in Figure 3. The results of correlation analysis are illustrated in Table 2. There was merely a moderate correlation between qCS (M2) and vCS; while, qCS (total) and qCS (M3-distal) had substantial correlation with vCS (*r* = 0.78, *r* = 0.76, respectively; all *P* < 0.001). And qCS (M3-distal) produced a little higher agreement with the consensus vCS than qCS (total) did [ICC: 0.69 (0.52–0.80) vs. 0.50 (0.28–0.68), all *P* < 0.001]. Although the reliability was of only moderate degree between qCS and vCS, the agreement for identifying favorable collateral scores (Tan score ≥ 2) compared to consensus were 97.56% for qCS (M3-distal) and 92.68% for qCS (total), respectively (Figure 4).

**TABLE 2:** Correlation between vCS and qCS, collateral scores and mRS.

	qCS (total)	qCS (M3-distal)	qCS (M2)	qCS (M1)	vCS
vCS	0.78	0.76	0.51	0.21	–
	*P* < 0.001*	*P* < 0.001*	*P* < 0.001*	*P* = 0.131	–
mRS	–0.43	–0.50	–0.22	–0.06	–0.47
	*P* = 0.001*	*P* < 0.001*	*P* = 0.108	*P* = 0.646	*P* < 0.001*

*Statistically significant.

vCS, visual collateral score; qCS, quantitative collateral score; mRS, modified Rankin Scale.

### Quantitative collateral score (M3-distal) is superior over visual collateral score in predicting clinical outcome

The Spearman correlation analysis implied that qCS (M3-distal) correlated a slightly stronger with 90-day mRS than vCS did (*r* = –0.50, *P* < 0.001; *r* = –0.47, *P* < 0.001; respectively) (Table 2). The predictive power of qCS (M3-distal) was superior to that of vCS [qCS (M3-distal): area under the receiver-operating characteristic curve (AUC), 0.81 (0.69–0.92), *P* < 0.001; vCS: AUC, 0.74 (0.61–0.87), *P* = 0.003] with a higher sensitivity [qCS (M3-distal) vs. vCS: 72.7% vs. 40.9%]. And the optimal cutoff point was 88.29% (72.7% sensitivity, 81.8% specificity) with qCS (M3-distal), and 1.5 (95.5% sensitivity, 39.4% specificity) with vCS.

### Identification of outcome-related risk factors

Based on the results of agreement and correlation analysis, qCS (M3-distal) rather than qCS (total) was preferred. Age, baseline NIHSS score, glucose levels, LDL-C levels, and collateral circulation were statistically significant in the univariate analysis (Supplementary Table 1). These factors, then, were integrated into the multivariable logistic regression models. The analysis showed that qCS, vCS, LDL-C, and age were independent predictors of clinical outcome receiving MT (Table 3). After adjusting for LDL-C and age, a 1% increase in qCS (M3-distal) corresponded to a 10% better outcome of patients with AIS. The adjusted odds ratio (OR) for vCS in predicting favorable outcome was 3.36.

**TABLE 3:** Identification of outcome-related risk factors using multivariable regression analysis.

Factors	Odds ratio (95% CI)	*P*-value
**Model 1**		
Age per 1 year	0.93 (0.86–1.00)	0.044*
Baseline NIHSS per 1 point	0.95 (0.84–1.07)	0.403
Glucose per 1 mmol/L	0.82 (0.58–1.15)	0.253
LDL-C per 0.1 mmol/L	1.15 (1.04–1.28)	0.010*
vCS per 1 point	3.36 (1.07–10.56)	0.038*
**Model 2**		
Age per 1 year	0.92 (0.84–1.00)	0.048*
Baseline NIHSS per 1 point	0.93 (0.81–1.07)	0.305
Glucose per 1 mmol/L	0.80 (0.52–1.21)	0.288
LDL-C per 0.1 mmol/L	1.20 (1.06–1.37)	0.004*
qCS (M3-distal) per 1%	1.10 (1.03–1.18)	0.007*

*Statistically significant.

CI, confidence interval; NIHSS, National Institutes of Health Stroke Scale; LDL-C, low-density lipoprotein cholesterol; vCS, visual collateral score; qCS, quantitative collateral score.

### Prognosis prediction model

The performances of different models are presented in Table 4. Herein, qCS (M3-distal) and vCS predicted favorable outcomes with accuracy of 78.2 and 69.1%, respectively. And the models based on qCS (M3-distal) to measure collaterals are generally more sensitive than those models based on vCS. Although the largest AUC value of 0.91 was achieved in the model of a combination of qCS (M3-distal), LDL-C and age, the accuracy was just increased slightly with 3.6% compared to that of qCS (M3-distal) alone.

**TABLE 4:** Performance metrics of the different models based on qCS or vCS.

Models	AUC (95% CI)	*P-*value	TPR, %	TNR, %	ACC, %
qCS (M3-distal)	0.81 (0.69–0.92)	<0.001	72.7	81.8	78.2
qCS (M3-distal) + LDL-C	0.88 (0.79–0.97)	<0.001	84.8	72.7	80.0
qCS (M3-distal) + age	0.82 (0.71–0.93)	<0.001	78.8	59.1	70.9
qCS (M3-distal) + LDL-C + age	0.91 (0.83–0.99)	<0.001	72.7	87.9	81.8
vCS	0.74 (0.61–0.87)	0.003	40.9	87.9	69.1
vCS + LDL-C	0.81 (0.69–0.93)	<0.001	68.2	81.8	76.4
vCS + age	0.80 (0.68–0.91)	<0.001	50.0	78.8	67.3
vCS + LDL-C + age	0.86 (0.77–0.96)	<0.001	72.7	84.8	80.0

qCS, quantitative collateral score; vCS, visual collateral score; AUC, area under curve; CI, confidence interval; TPR, true positive rate; TNR, true negative rate; ACC, accuracy; LDL-C, low-density lipoprotein cholesterol.

## Discussion

In this study, we identified that collateral scores, LDL-C and age were independent preoperative predictors of the outcome of patients with AIS receiving MT and demonstrated that qCS performed better than vCS (Tan score) in predicting the clinical outcome.

Many predictors of the clinical outcome of patients with AIS receiving MT have been reported so far, including age, stroke severity, 24-h blood pressure after MT, 24-h NIHSS score after interventional therapy, and final infarct volume (Cernik et al., 2019; Wollenweber et al., 2019; Brugnara et al., 2020). However, some of these factors are postoperative indicators that cannot help with or provide timely advice before clinical decision-making. Our study reported that higher collateral scores, higher LDL-C and younger age were the independent preoperative predictors for a better neurologic outcome. It has been widely accepted that LDL-C has an atherogenic effect on large vessels; however, the protective effect of hyperlipidemia on clinical outcome in patients with AIS is not widely acknowledged. Cuadrado-Godia et al. (2009) reported that higher total cholesterol and LDL-C levels were associated with better outcomes in male patients after the first AIS. There could be underlying mechanisms to this effect: antioxidation and endothelial protection, namely neutralizing free radicals and down-regulation of the vascular endothelial growth factor, respectively (Yeramaneni et al., 2017). Higher but within the normal range LDL-C was also found in the 90-day favorable outcome group in our current study, which adds to the existing evidence of the protective effect of lipid paradox (Cheng et al., 2018). Another prognostic factor with statistical significance in the logistic regression model was age, which was consistent with previous studies that younger age was associated with a good outcome with OR 1.06 (Wollenweber et al., 2019; Yao et al., 2020).

Our study also confirmed that patients with AIS having good collaterals would have lesser neurological impairment, which was in line with many previous studies (Berkhemer et al., 2016; Anadani et al., 2021; Uniken Venema et al., 2022). And a patient with a qCS (M3-distal) of < 88.29% would be predicted to have an ominous prognosis, with a cutoff higher than the cutoff vCS (50%) (Lu et al., 2019), even if the tissue perfusion parameters met the criteria for MT. In predicting clinical outcome, qCS (M3-distal) is superior over vCS with a higher AUC value and a higher sensitivity. The reason for the discrepancy may lie in the nature of evaluation methods, namely quantitative method vs. categorical method. The conventional vCS systems, which are based on traditional or dynamic CTA, depend on manual visual assessment, making them prone to both intra- and inter-observer variations (McVerry et al., 2012; Aktar et al., 2020; Cui et al., 2021). Several vCS systems have been proposed. The prognostic power of different scoring systems varied in different studies, and was far from satisfactory with AUCs ranging from 0.59 to 0.77 (Berkhemer et al., 2016; Lu et al., 2019; Seker et al., 2020; Cui et al., 2021). No consensus has been reached yet in terms of the evaluation criteria.

qCS is the ratio of the collateral volume on the affected side to that on the unaffected side, which quantifies collateral circulation and may reduce the effect of observers’ experience using visual methods. Boers et al. (2018) acquired qCS using Hessian-based enhancement filters and revealed that qCS had substantial correlation with vCS (*r* = 0.75, *P* < 0.001), a similar result with our study (*r* = 0.78, *P* < 0.001). In addition, they also found that with an AUC of 0.71, qCS was slightly superior to vCS in predicting functional independence; but the difference was not statistically significant. In the subsequent study, a moderate ICC of 0.60 between qCS and vCS and an agreement of 81% in collaterals dichotomization were obtained in the agreement analysis (Wolff et al., 2022). Su et al. (2020) applied convolutional neural networks to quantify collateral circulation; the model achieved an accuracy of 80% in comparison to the vCS, and the value was increased to 90% for collaterals dichotomization. Similar results—a moderate ICC but a high agreement for identifying favorable collateral circulation (Tan score ≥ 2)—were also observed in our study. These interesting results revealed that the disagreement mainly occurred within the sets of Tan score 0 and 1 (0–50%) or Tan score 2 and 3 (50–100%). Recently, another quantitative measurement of the collateral circulation (V_CCq_) was proposed—the method calculating vessel volumes using the identified CT values at three selected layers on time maximum intensity projection (tMIP) CTA images (Cao et al., 2021). The study showed that both V_CCq_ and Tan score were moderately negatively correlated with final infarct volume; however, unlike Tan score, V_CCq_ was an independent predictive factor of the clinical outcome (OR = 0.14, *P* = 0.009). In addition, V_CCq_ had a better value than Tan score in predicting the clinical outcome [V_CCq_: AUC, 0.93 (0.85–1.00); Tan score: AUC, 0.79 (0.68–0.89); all *P* < 0.001], revealing that the quantitative method is superior to vCS in predicting the prognosis, which was also verified in our current study. The adjusted OR value (3.36) of vCS in this study was relatively too large, as 1 point increase in vCS corresponded to a 236% better outcome of patients with AIS. The large span, also found in a recent study (Uniken Venema et al., 2022), reinforced the point that vCS was inferior to qCS due to its unrefined categorical method.

Although a statistically significant difference in baseline NIHSS score and admission glucose levels was observed between favorable and unfavorable outcome groups, neither of them could predict clinical outcomes. Hyperglycemia on admission in patients with AIS was usually transient and frequently caused by temporary stress (Ntaios et al., 2011). In our current study, post-stroke hyperglycemia (defined as admission glucose levels ≥ 7.8 mmol/L; Zuurbier et al., 2016; Zonneveld et al., 2017) may have mainly resulted from stress as only thirteen patients had a history of diabetes mellitus. Tziomalos et al. (2017) reported that stress hyperglycemia was not an independent predictor of poor outcome but was rather associated with a more severe stroke (admission NIHSS). In regard to NIHSS score, early neurological change or the change of NIHSS score, rather than baseline score, was significantly and independently associated with 90-day outcomes (Heitsch et al., 2021. While, some studies found that a lower baseline score was associated with a favorable outcome (Sojka et al., 2021) and the performance of NIHSS score in predicting functional outcome was time-dependent (Wu et al., 2019). Therefore, the prognostic prediction power of glucose levels and baseline NIHSS score remains controversial.

Numerous attempts have been made to accurately predict the AIS treatment outcome, and several stroke outcome prediction scores have been developed to help physicians make better therapy decisions; however, difficulties and challenges still exist. Brugnara et al. (2020) developed a machine learning-based predictive model with an AUC score of 0.86; however, it combined twelve input variables from clinical, imaging, and angiographic aspects and was inconvenient for rapid clinical evaluation. Conversely, even though the present study had only three variables involved in the prediction model, namely qCS (M3-distal) + LDL-C + age, our model offered strong prognostic prediction power with an AUC value of 0.91, which was better than that of stroke outcome prediction scores (AUC 0.70–0.86) (Matsumoto et al., 2020). Furthermore, qCS alone could also predict favorable outcome with an AUC value of 0.81 and an accuracy of 78.2%. Although the V_CCq_ mentioned above had a superior prognostic value for clinical outcome than qCS (M3-distal), the parameter was derived from CTA images of 19 phases, which were not available for some hospitals. Our model, from the single-phase CTA, is simple and effective and has the potential to be applied in the acute clinical setting.

Our study had some limitations. First, artificial bias may be introduced in the annotation of collateral vessels. To minimize bias, repeated training and practice were conducted before annotation until the approval from the senior neuroradiologist. Second, the sample size was small at only 55 cases; however, the power values of our study were above 0.8 (ranging from 0.81 to 1.00), suggesting that the results obtained with the present sample size were reliable (Serdar et al., 2021). Prospective multicenter studies will be conducted in the future to further validate the results and address the issue of potential data bias. Although, in present study, the vessels segmentation is labor- and time-consuming which is not suited for the scenario of acute stroke management now, our results prove that collateral quantification is feasible and also widen the application range of artificial intelligence in the future.

## Conclusion

Collateral scores, LDL-C and age were independent prognostic predictors in patients with AIS receiving MT, and the performance of qCS (M3-distal) was superior to that of vCS. Furthermore, qCS + LDL-C + age offers a strong prognostic prediction power and qCS (M3-distal) alone was another good choice for predicting clinical outcome.

## Data availability statement

The data that support the findings of this study are available from the corresponding authors upon reasonable request.

## Ethics statement

The studies involving human participants were reviewed and approved by the Ethics Committee of Huashan Hospital. Written informed consent for participation was not required for this study in accordance with the national legislation and the institutional requirements.

## Author contributions

QL: conceptualization, methodology, investigation, data curation, formal analysis, and writing—original draft. HZ and JF: methodology, investigation, and data curation. XC: methodology, investigation, and formal analysis. YP: resources and formal analysis. XZ: investigation and data curation. JW: conceptualization, investigation, and data curation. DG: resources, writing—review and editing, supervision, and funding acquisition. JZ: conceptualization, formal analysis, writing—review and editing, supervision, and funding acquisition. All authors contributed to the article and approved the submitted version.

## Footnotes

1
http://www.itksnap.org

## Abbreviations

MT: mechanical thrombectomy
AIS: acute ischemic stroke
CTA: computed tomography angiography
vCS: visual collateral score
qCS: quantitative collateral score
LDL-C: Low-density lipoprotein cholesterol
mRS: modified Rankin Scale
MCA: middle cerebral artery
ICA: internal carotid artery
CTP: computed tomography perfusion
NCCT: non-contrast computed tomography
NIHSS: National Institute of Health stroke scale
TOAST: trial of ORG 10,172 in acute stroke treatment
mTICI: the modified Treatment In Cerebral Ischemia
ICC: intraclass correlation efficient
ROC: receiver operating characteristic
AUC: area under the curve
OR: odds ratio.
