# Supplementary material for: Quantitative collateral score for the prediction of clinical outcomes in stroke patients: Better than visual grading
Source: Front Neurosci. 2022 Oct 25;16:980135. doi: 10.3389/fnins.2022.980135 (PMC9641373; doi:10.3389/fnins.2022.980135)
Supplement: Supplementary file 1 [file Data_Sheet_1.PDF]

Table A online supplement. Results of univariable regression analysis

Characteristics	Odds Ratio (95% CI)	*P* value
Age, years	0.949 (0.904–0.995)	0.030
Male sex	1.073 (0.335–3.439)	0.905
TOAST type	0.536 (0.243–1.183)	0.122
Baseline NIHSS	0.886 (0.788–0.996)	0.042
Platelet, 10^9/L	1.008 (0.995–1.021)	0.225
INR	1.026 (0.017–60.423)	0.990
Glucose, mmol/L	0.754 (0.551–1.030)	0.076
LDL-C, mmol/L	2.630 (1.142–6.054)	0.023
vCS (Tan score)	4.646 (1.709–12.631)	0.003
qCS(M3-distal), %	1.074 (1.028–1.121)	0.001
Infarct core, mL	0.965 (0.924–1.007)	0.103
Ischemic volume, mL	0.998 (0.987–1.009)	0.998
Mismatch volume, mL	1.000 (0.990–1.011)	0.947

Abbreviations: CI, confidence interval; TOAST, trial of ORG 10172 in acute stroke treatment; NIHSS, Nation Institutes of Health Stroke Scale; INR, international normalized ratio; LDL-C, low-density lipoprotein cholesterol; vCS, visual collateral score; qCS, quantitative collateral score
